# Magnetic resonance imaging safety‐driven, heat‐mitigating design for a magnetic resonance imaging‐guided, magnetically actuated robotic catheter

**DOI:** 10.1002/mp.70630

**Published:** 2026-08-10

**Authors:** E. Erdem Tuna, Gabriel J. Foss, Sanjana Kamath, Ridaa Z. Ali, Yen‐Chun Chen, Ahmed B. Aydin, Reid Bolding, Mark Griswold, M. Cenk Cavusoglu

**Affiliations:** ^1^ Department of Electrical, Computer, and Systems Engineering Case Western Reserve University Cleveland Ohio USA; ^2^ Department of Physics Case Western Reserve University Cleveland Ohio USA; ^3^ Department of Radiology Case Western Reserve University Cleveland Ohio USA

**Keywords:** interventional MRI device safety, MRI‐guided robotic catheter, MRI RF heating

## Abstract

**Background:**

Metallic components in magnetic resonance imaging (MRI)−guided robotic catheters, such as leadwires and microcoils, can interact with the scanner's radiofrequency (RF) fields, causing heating via the antenna effect and Joule heating, leading to localized temperature rises and an increased risk of thermal injury. Ensuring thermal safety will enable safe and reliable operation of magnetically actuated robotic catheters within MRI environments.

**Purpose:**

This study aims to develop a novel MRI‐compatible robotic catheter design that mitigates RF‐induced and resistive heating, ensuring patient safety and proper functionality during MRI‐guided interventions, particularly for cardiac ablation procedures. The catheter is directly actuated by magnetic torques generated on current‐carrying microcoils at the tip by the MRI scanner's static magnetic field, allowing precise navigation.

**Methods:**

The catheter design incorporates systematically tuned microcoil parameters and distributed capacitors along the leadwires to suppress resonance and minimize RF energy absorption. A dithering technique applying actuation currents at a 50% duty cycle (10 Hz, 50 ms on/off) promotes forced convection to reduce resistive heating. A resistor‐capacitor (RC) equivalent circuit model was used to characterize the catheter's thermal behavior. Thermal validation was conducted on a 3T MRI scanner using a saline‐filled phantom, fiber‐optic temperature probes, and a balanced steady‐state free precession (bSSFP) imaging sequence with actuation currents up to 1 A. Hot spot detection sweeps along the leadwires and spatial heating assessments at multiple bore positions were also performed.

**Results:**

The prototype exhibited a maximum temperature increase of 0.6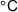 during simultaneous imaging and 1 A dithered actuation, well within Food and Drug Administration (FDA) safety limits. No hot spots were detected along the leadwires, and thermal performance remained consistent across all bore positions tested.

**Conclusions:**

The catheter design effectively mitigates both RF‐induced and resistive heating under the tested conditions. These results demonstrate the suitability of the design for MRI‐guided interventional procedures under the tested conditions, while remaining within FDA temperature limits.

## 1 INTRODUCTION

This work presents the magnetic resonance imaging (MRI) safety‐driven thermal design and validation of a magnetically actuated, MRI‐guided robotic catheter. Specifically, this paper focuses on mitigating radiofrequency (RF)‐induced and resistive (Joule) heating in a full‐length device, presenting design and RF response tuning of magnetic actuation coils and actuation dithering for thermal management. Mechanical design, actuation, and imaging performance of MRI‐actuated steerable catheters have been investigated in,^1^, ^2^, ^3^ and imaging performance is described in the study by Ali et al.^4^ As such, the present study is limited to thermal safety analysis and does not address mechanical performance, imaging performance, or clinical validation, which are beyond its scope. Clinical translation and in vivo evaluation remain the subject of ongoing and future work.

The catheter integrates distributed capacitors and coil detuning to suppress resonance near the scanner's Larmor frequency, together with a dithering‐based convection method that applies actuation currents at a 50% duty cycle to promote heat dissipation. These design features were evaluated through RF reflection ($S_{11}$) characterization, thermal‐equivalent circuit modeling, and phantom‐based temperature measurements using fiber‐optic probes. The measured thermal response was benchmarked against FDA temperature‐rise limits, confirming compliance under imaging and actuation conditions.

Existing standards (ISO/TS 10974 for active implantable devices and ASTM F2182 for passive implants) do not apply to partially implanted or externally powered, patient‐contacting systems, leaving no standardized procedure for assessing RF heating in MRI‐actuated catheters. To address this gap, a custom phantom and validation protocol were developed to replicate the conductive and convective environment of intracardiac use while allowing simultaneous imaging and actuation. This configuration enables controlled, repeatable assessment of RF‐induced and resistive heating under realistic MRI conditions, using a saline‐doped water medium that provides tissue‐equivalent loading representative of in vivo conductivity and permittivity.

### 1.1 Background and clinical needs

MRI‐guided interventions are increasingly being used in clinical settings because of MRI's excellent soft tissue contrast and radiation‐free imaging capabilities.^5^, ^6^ Various intraoperative MRI‐guided electromagnetically actuated catheter designs have been explored.^7^, ^8^, ^9^, ^10^, ^11^, ^12^, ^13^, ^14^ One key application is cardiac catheter ablation, a common treatment for arrhythmias such as atrial fibrillation (AFib). However, automated catheter navigation in the MRI environment remains challenging due to limited real‐time imaging speed, restricted access within the scanner bore, and the need for MR‐compatible actuation and tracking.^15^

This work considers an MRI‐guided, magnetically actuated steerable catheter system^1^, ^2^, ^16^, ^17^ designed for such procedures. The catheter incorporates current‐carrying microcoils at its tip that generate magnetic torques within the MRI scanner's static $B_{0}$ field, enabling controlled tip deflection managed remotely from the scanner control room. MRI‐actuated steerable catheters have been characterized in terms of mechanical and actuation performance,^1^, ^2^, ^3^ including trajectories mimicking circumferential and linear ablation paths used in atrial fibrillation procedures.^18^ The catheter construction and coil configuration are described in Section 2.

### 1.2 Heating challenges

Metallic elements within the catheter, such as the leadwires, actuation coils, and imaging coils, can interact with the MRI scanner's RF field, potentially leading to temperature increases across various sections of the device due to resonating RF waves. The leadwires connecting actuation coils to the power supply can cause unwanted heating due to coupling with the RF fields, a phenomenon known as the antenna effect.^19^, ^20^ Additionally, electric currents necessary to produce the required magnetic moments for catheter steering can result in Joule (resistive) heating, potentially raising temperatures to unsafe levels.

The resulting heating in the microcoils and leadwires can raise the specific absorption rate (SAR) in surrounding tissues, potentially leading to excessive localized temperature increases and causing burns.^21^ The U.S. Food and Drug Administration (FDA) advises that temperature increases due to the SAR should not surpass 1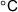 within the head, 2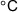 within the torso, and 3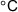 within the extremities.^22^

### 1.3 Prior work and limitations

RF‐induced heating of intravascular devices in MRI has been studied extensively. Early work by Konings et al.^23^ demonstrated rapid temperature increases at guidewire tips due to RF resonance, and Ladd^19^ proposed coaxial chokes to mitigate energy absorption along catheter lengths. Subsequent approaches to interrupt resonance modes along leadwires include distributed transformer‐based segments^24^, ^25^ and miniature tethered RF traps that suppress heating across multiple conductors with a single device.^26^ Complementary work has examined the influence of device position inside the scanner and lead geometry on heating magnitude,^27^, ^28^ while Yeung et al.^29^ introduced a safety index for systematic risk assessment.

For catheter‐mounted actuation coils specifically, Settecase et al.^30^ studied RF heating in steering coils, and Bernhardt et al.^31^ showed that optimized coil materials and geometries can reduce resistive heat generation. Reiter et al.^32^ examined whether tip irrigation can enhance catheter safety during cardiovascular MR procedures. More recently, Phelan et al.^33^ proposed a heat‐mitigated Lorentz‐force microcatheter for MRI‐guided surgery.

Several prior studies^1^, ^2^, ^14^, ^17^ investigated Lorentz‐torque catheter actuation using short prototypes approximately six inches in length, which do not replicate the heating challenges posed by full‐scale systems. Full‐size catheters^30^, ^31^, ^33^ often rely on single‐layer, laser‐lithographed coils that limit torque due to excessive heat from high‐current operations. Prior solutions have focused primarily on RF suppression using distributed components, without addressing Joule heating from actuation currents.

### 1.4 Proposed approach and contributions

The present work extends earlier research^34^ by addressing both RF and Joule heating effects together in a novel full‐length catheter prototype. The design integrates three complementary strategies: (1) capacitor placement to break resonance modes, (2) coil detuning to minimize absorption at scanner frequencies, and (3) dithering‐driven forced convection to reduce Joule heating. The impact of coil parameters on the RF response is studied and used to guide a heat‐mitigating design process by shaping the reflection coefficient^35^ of the microcoils to prevent resonance near the Larmor frequency. The thermal response is analyzed using an equivalent circuit model.

An actuation dithering technique applies currents at a 50% duty cycle at 10 Hz to dissipate heat generated by the microcoils through forced convection. This mechanism operates at high frequency and low amplitude, does not introduce abrupt or unstable motion, and does not interfere with the intended trajectory. The dithering actuation mechanism was used as the primary mode of deflection and was sufficient to produce stable catheter tip displacement.

## 2 METHODS

**FIGURE 1:**
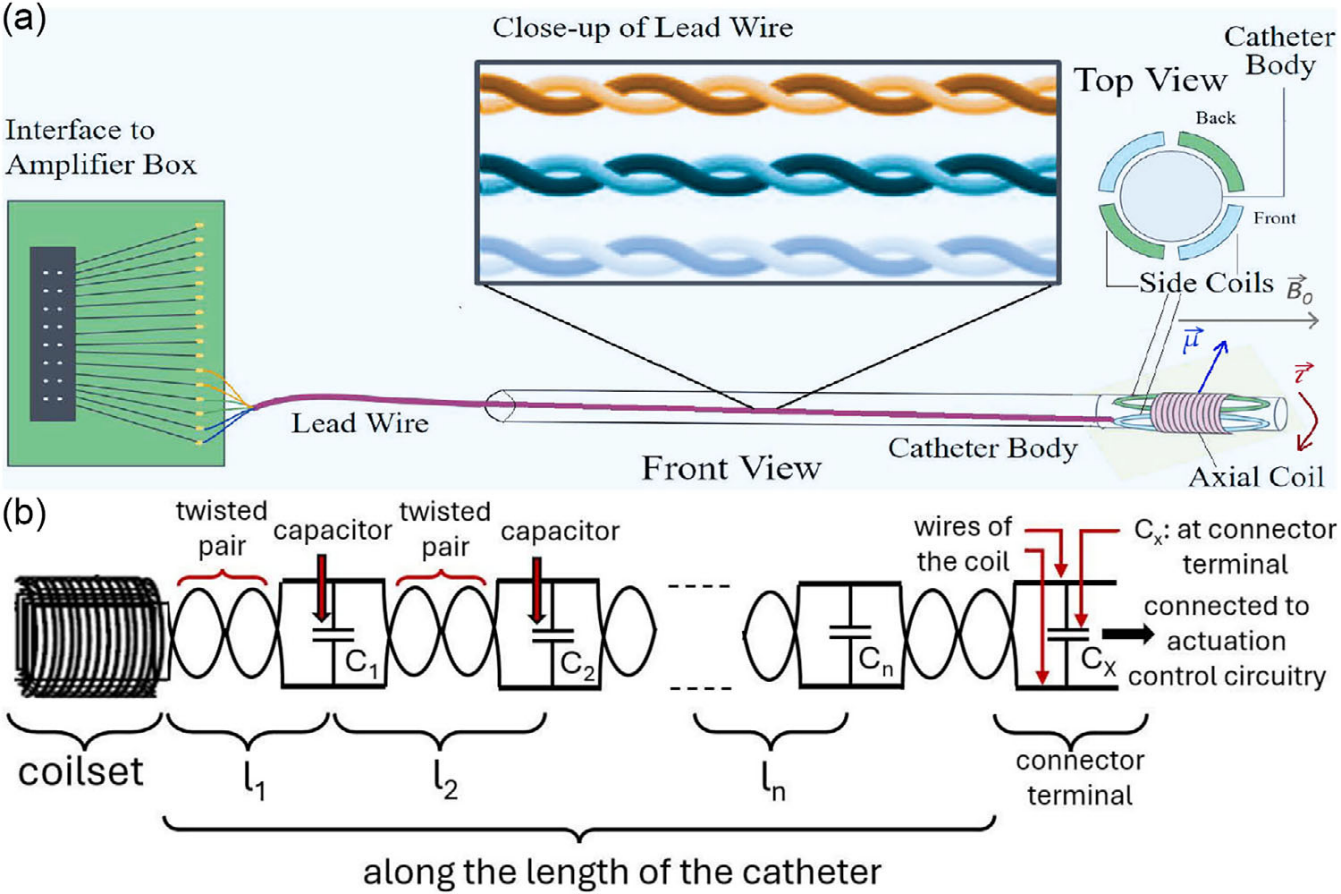
Overall structure of an MRI‐actuated steerable catheter prototype. (a) The catheter is equipped with a single coil set. Each coil set includes one axial coil and two orthogonal transverse (side) coils. The leadwires consist of tightly wound, twisted pairs of wires, one pair for every coil in the coil set. The magnetic torque τ=μ×B acting on the current‐carrying coils within the scanner's static field is illustrated at the catheter tip. (b) Capacitors are placed in parallel along the catheter leadwires.

The catheter prototype consists of the catheter body, one or more actuation coil sets, and leadwires, as shown in Figure 1a.^1^, ^3^, ^34^ Each coil set on the catheter is composed of three orthogonal coils: one axial and two transverse (side) coils. The leadwires are tightly twisted together to minimize electromagnetic coupling with the MRI scanner. The proximal end of the leadwires is terminated on a circuit board that connects to the current driver unit, which functions as the amplifier supplying current to the actuator coils. Coils have separate channels so that they can be actuated independently of one another. The torque induced on a current‐carrying coil in a magnetic field is given by (Figure 1a):

(1) τ=μ×B

where τ is the induced torque, μ is the magnetic moment vector of the coil, and B is the external magnetic field vector. The magnetic moment vector μ is:

(2) $$\mu_{j}=N_{j}A_{j}i_{j}$$

where $N_{j}$ is the number of winding turns of coil j, $i_{j}$ is the current passing through coil j, and $A_{j}$ is the vector area of coil j with the vector direction given by the right‐hand rule.

### 2.1 RF considerations for the catheter prototype

#### 2.1.1 Catheter actuation coil set

The reflection coefficient $S_{11}$ of a set of coils represents the amount of RF energy reflected back from the coils relative to the incident energy applied and can be a useful guideline for identifying the frequencies at which resonances occur.^36^ If a coil set with a low reflection coefficient at or around the Larmor frequency of the scanner^37^ is exposed to the RF field of the MRI, it will absorb an excessive amount of power from the MR scanner. The coils may induce unintended currents within the MR system and potentially overload the circuitry of the current driver unit that supplies actuation currents, increase the SAR of surrounding tissues, and generate undesirable amounts of heat through RF heating.

In this study, the reflection coefficients of various candidate coil sets were measured to guide the coil design and tuning process. The coil set with no observable resonances in the vicinity of the Larmor frequency and its subsequent two harmonics was selected for further thermal testing to ensure that the temperature increase remained below 2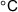, as specified by FDA guidelines.^22^ For the 3T MR scanner used in this study,^1^ the Larmor frequency is 123 MHz, and the two subsequent harmonics of concern are 246 MHz and 369 MHz.

#### 2.1.2 Catheter leadwires

The long catheter leadwires can act as resonant antennas when their length approaches a quarter‐ or half‐wavelength of the MRI Larmor frequency, leading to substantial coupling with the RF field. Such resonance can create standing‐wave patterns and localized hot spots, posing safety risks due to the antenna effect.

Capacitors are used to mitigate RF‐induced heating by disrupting resonance conditions and modifying impedance along the leadwires. Capacitors are placed along the catheter leadwires (Figure 1b) with non‐uniform spacing, specified in the Methods, to reduce coupling with the transmit field. This arrangement shifts resonant frequencies away from the Larmor frequency, minimizes RF‐induced currents, and prevents localized heating. The capacitors also create impedance mismatches that further reduce RF coupling and temperature rise along the leadwires.^34^

### 2.2 Coil set design and RF response tuning

#### 2.2.1 Design variables for the coil set

The design of the actuation coils is driven by a need to meet physical dimension constraints, generate sufficient magnetic torque to achieve desired catheter motions and contact forces, the coil set configuration, and the RF characteristics of the coils. This section examines the impact of the configuration of the coil set on the resulting RF response, and tunes these responses to make the catheter coils reliable and suitable for MRI applications. The coil configuration is determined by the wire gauge ${w_{g}}_{i}$, the winding turn number of coils $N_{i}$, and the number of axial coil layers $n_{l}$, which are summarized in Table S1.

Building on previously established torque and size constraints,^3^ the present study focuses on coil tuning and RF characterization to suppress resonance near the Larmor frequency while preserving the magnetic‐torque capability required for catheter steering. Results from earlier work^3^ indicate that the coil dimensions should not exceed 4 mm (12 Fr) in diameter and 10 mm in length due to clinical size limits, and that a magnetic moment of at least 0.34 A· $m^{2}$ is needed for effective atrial‐fibrillation ablation tasks.

**FIGURE 2:**
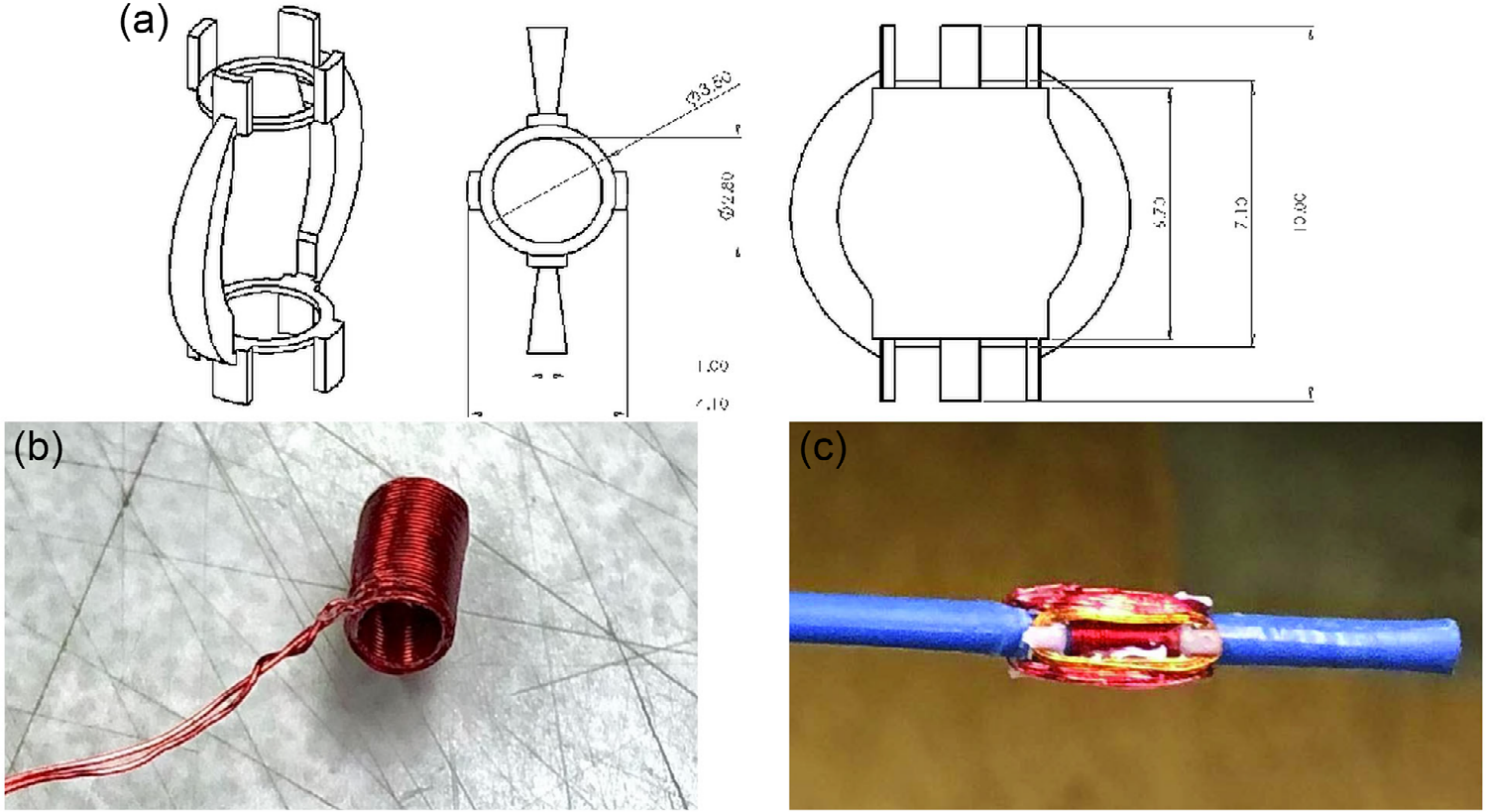
(a) Bobbin schematics are shown with dimensions given in mm. Once all wires are wound around the bobbin, cyanoacrylate glue encapsulates the prototype, and the wing‐like supports of the bobbin are removed. (b) An axial coil without the bobbin and transverse coils. (c) An assembled coil set on the catheter prototype with leadwires contained inside the catheter lumen.

In order to meet these dimension constraints, a novel minimal bobbin profile is used in this study. The bobbin was manufactured from FormLabs Rigid 10K resin using an Elegoo Mars 3 LCD printer. The coil set slots into the bobbin, the bobbin slides into the tip of the catheter, and is then glued into place on the catheter tubing. The custom coils have been wound by hand on a precision coil winding machine. The bobbin schematics showing its dimensions, an axial coil, and a sample coil set assembled on a catheter prototype can be seen in Figure 2a–c.

The bobbin was designed with a minimal profile, allowing more dynamic adjustments to the axial coil compared to the transverse coils, which have limited tuning flexibility due to space constraints around the bobbin cleats. Therefore, tuning of the axial coil is prioritized.

#### 2.2.2 Design of the coil set

A variety of wire gauges between 22 and 38 AWG are considered for coil design. The wire gauge defines both the wire thickness and current‐carrying capacity (ampacity) of the wires. These in turn affect the magnetic moment of the coils (2), as the wire thickness alters the cross‐sectional area of the coils as well as the number of winding turns and number of layers that could be fitted on the bobbin, whereas ampacity determines the maximum current that coils can carry safely.

The number of coil layers that fit on the bobbin given the dimension constraints (Figure 2a) for a specific wire gauge can be determined by:

(3) $$n_{l}=\max\left\{n\in Z\;|\;n\leq \frac{w_{e}}{d_{w}}\right\},$$

where $w_{e}$ is the effective bobbin enclosure width and $d_{w}$ is the wire diameter. For a catheter tubing with a 2.6 mm diameter (Figure 2a) and a 4 mm total width limit,^3^ the remaining 1.4 mm allows 0.7 mm of wire coil on each side. To account for practical considerations and manufacturing tolerances, 80% of this width is used as the working dimension, resulting in an effective enclosure width of 0.56 mm.

The diameter and ampacity of various wire gauges, and the corresponding number of coil layers computed from (3) are given in Table S2.

The maximum number of winding turns is determined by the physical dimension constraints:

(4) $${N_{a}}_{max}=\max\left\{N\in Z\;|\;N\leq \frac{l_{e}}{d_{w}}\times n_{l}\right\},$$

where $l_{e}$ is the effective bobbin enclosure length. The middle of a coil may be tightly wound, but the lack of support at the bobbin ends results in uneven windings. With a bobbin enclosure length of 6.7 mm (Figure 2a), a 0.9 packing factor is applied to account for this nonuniform density, yielding an effective enclosure length of 6.03 mm.

The minimum number of winding turns is determined by the minimum magnetic moment required to perform cardiac ablation tasks safely.^3^ From (2):

(5) $${N_{a}}_{min}=\min\left\{N\in Z\;|\;N\geq \frac{\mu }{i_{w}A}\right\},$$

where $A=\pi {\left(r_{w}n_{l}\right)}^{2}$ with $r_{w}$ is the wire radius, as the axial coil (Figure 1) has a circular cross section, and $i_{w}$ is the wire ampacity (Table S2).

The maximum and minimum number of winding turns are computed respectively from (4) and (5) for the wires summarized in Table S2. The resulting coil space is shown in Figure S1.

#### 2.2.3 RF response of the coil set

Theoretically modeling and accurately calculating the RF response of the coils is very difficult due to their complex geometry, geometric inconsistencies, interwinding capaci‐ tance, and the influence of leadwire length on coil behavior. Consequently, the reflection coefficient $S_{11}$ was measured experimentally to evaluate the RF response and guide the coil design process. In these measurements, a magnetic coupling probe was brought into close proximity with the coil set, inductively transferring RF energy from the network analyzer to the coils to measure their resonant response.

A probe with a 4‐turn, 6 mm loop was constructed to couple into the coil set and then used with the network analyzer.^2^ The design of the probe follows the implementation described by Kamath et al.^34^ and the probe is shown in Figure S2. The network analyzer sends an input signal through the probe, while the measurement coil at the end of the probe is carefully swept over the catheter surface and around the coil set. This forms an electromagnetic linkage, allowing the analyzer to measure and display the reflected signal from the probe. The reflection signal varied slightly as the probe was swept across the coil set; therefore, the lowest reflection coefficient (i.e., the maximum RF coupling) observed during the sweep was recorded to represent the worst‐case response for each coil configuration.

#### 2.2.4 Tuning the coil set

To minimize RF coupling, actuator coils were detuned using their intrinsic self‐resonance. $S_{11}$ measurements were performed at the coil terminals with the transmission line and parallel capacitors in place. These capacitors act as a distributed low‐pass filter, blocking RF while allowing pulsed currents for actuation. Inductance values for the wire gauges are listed in (Table S3).

After analyzing the RF response results, the winding turns of both 33 AWG axial and 36 AWG transverse coils were maximized within the bobbin dimensions constraints to shift the resonant peaks away from the Larmor frequency. Improved coil manufacturing led to better tolerances, yielding the final turn counts shown in Table 1.

**TABLE 1:** Final turn counts of the catheter coils. Corresponding coil wire gauges are also given.

Coil type	Turn count	Total	Coil Wire Gauge (AWG)
X Transverse Coil	18 + 18	36	36
Y Transverse Coil	18 + 18	36	36
Two Layer Axial	29 + 27	56	33

The RF wavelength of the MR scanner in free‐space at 123 MHz Larmor frequency is:

(6) $$\lambda =\frac{v}{f}=\frac{3\times 10^{8}\;\text{m/s}}{123\times 10^{6}\;\text{Hz}}=2.43m.$$

**FIGURE 3:**
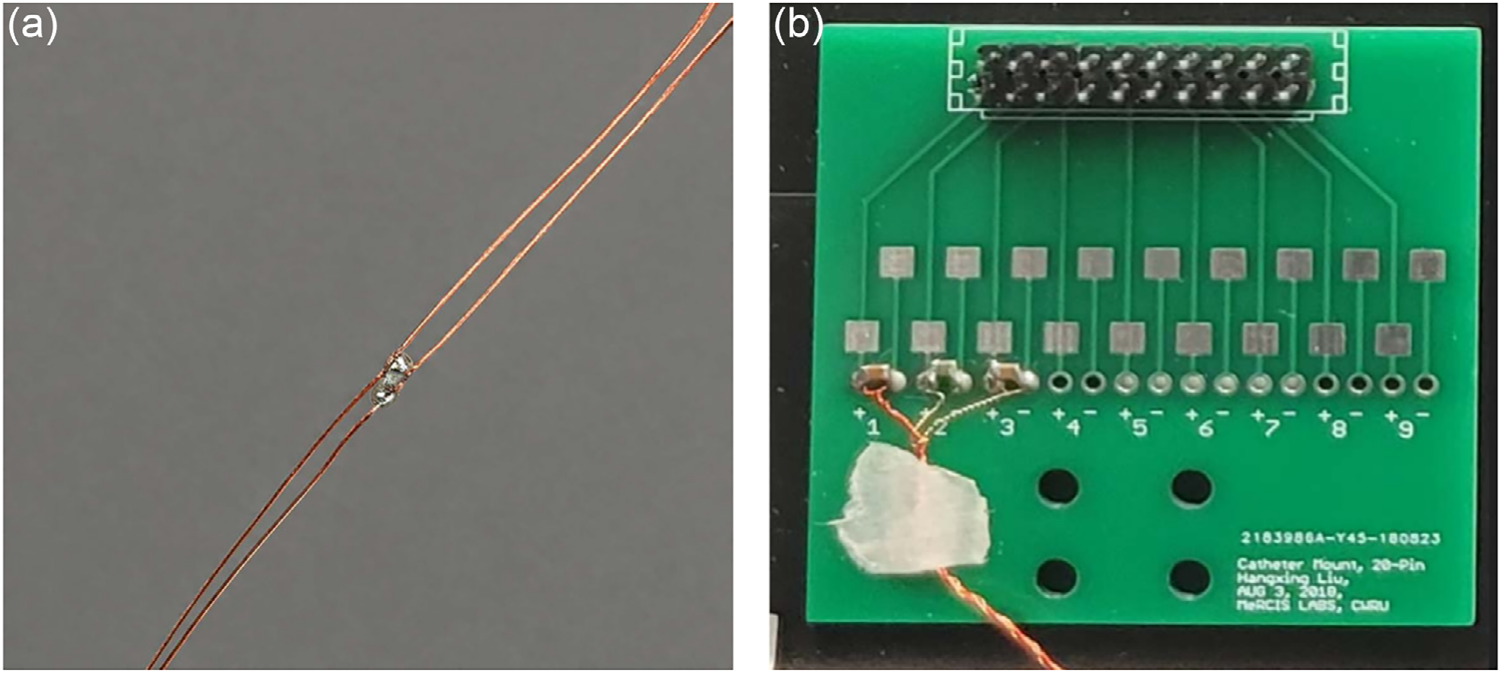
(a) Leadwire capacitor. The untwisted wires were configured into a twisted pair to further minimize electromagnetic interference. (b) PCB capacitor.

Given that the lead wire supplying current to the catheter coils extends approximately 1.5 m in length, capacitors are strategically incorporated along the wire to mitigate RF absorption and minimize the antenna effect. Four 470 pF capacitors^3^ were placed along the leadwire from the bobbin to the proximal end of the catheter at intervals of 10, 20, 15, and 30 cm (Figure 3a). The capacitors were mounted on internal leadwires housed within the catheter's 2.1 mm inner diameter lumen, so the 2.6 mm outer diameter remains unchanged at these locations and the overall device profile is not affected. 0.1 μF 4 capacitors were placed on the PCB terminals of each of the three lead wires (Figure 3b). These capacitor values and non‐uniform spacing were empirically selected to tune the response to address potential resonance issues.^34^

The free‐space RF wavelength at the Larmor frequency is approximately 2.43 m (Equation 6), but would be considerably shorter within the body as a result of dielectric effects. Ideally, capacitors should be placed at approximately one‐quarter wavelength, which is around 60 cm in free space. While ideal capacitor placement for resonance suppression would typically consider this reduced wavelength, the catheter design used non‐uniform capacitor spacing ranging from 10 to 30 cm, intentionally chosen to disrupt regularly spaced standing wave patterns and prevent the formation of resonant conditions along the leadwires. Unlike controlled laboratory media, the human body is a highly heterogeneous dielectric environment, making the development of well‐defined standing waves even less likely. By avoiding uniform intervals, the capacitor distribution reduces the risk of constructive interference and localized RF heating.

### 2.3 Catheter thermal response model

**FIGURE 4:**
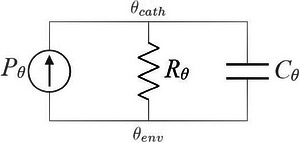
RC equivalent circuit model of the catheter's thermal response.

The heat generated by Joule (resistive) and RF heating is either dissipated into the surrounding environment or stored within the catheter, leading to an increase in temperature. Since the dissipated heat is governed by its thermal resistance $R_{\theta }$ and the stored heat by its heat capacity $C_{\theta }$, the catheter's thermal response can be modeled as an RC equivalent circuit as shown in Figure 4, where $P_{\theta }$ represents the total heat flow rate into the system, analogous to the current source, $\theta_{cath}$ and $\theta_{env}$ are respectively temperatures of the catheter coil set and the surrounding environment, analogous to voltage. The model is then described by:

(7) $$P_{\theta }=\frac{\Theta }{R_{\theta }}+C_{\theta }\frac{d\Theta }{dt},$$

where $\Theta =\theta_{cath}-\theta_{env}$ is the temperature difference between the coil set and the environment. The model incorporates the environment's temperature as a DC offset by subtracting the initial environment temperature during computation, ensuring the initial conditions $\theta_{cath}$(0) = $\theta_{env}$(0) = 0 and preserving linearity.

By defining total heat flow as the input and temperature difference as the output, the system's transfer function is given by:

(8) $$H\left(s\right)=\frac{\Theta (s)}{P_{\theta }\left(s\right)}=\frac{R_{\theta }}{sR_{\theta }C_{\theta }+1}.$$

Although the Joule heating and RF heating effects are intertwined, they can be separated for analyzing the system response:

(9) $$P_{\theta }=P_{J}+P_{RF},$$

where $P_{J}=i^{2}R_{coil}$ is the power generated due to Joule heating with i the actuation current and $R_{coil}$ the measured resistance of the coil, and $P_{RF}$ denotes the power generated from the absorbed RF energy.

This simplification is justified by the coil detuning described in Section 2.2.4, which suppresses RF energy absorption near the Larmor frequency. Prior studies on MRI catheter steering coils have separately characterized RF‐induced and resistive heating, finding that resistive heating from actuation currents is the dominant thermal effect,^30^ and that RF‐induced heating can be further suppressed through impedance design of the transmission lines.^25^ The validity of this assumption is confirmed experimentally in Section 3.2.2, where the temperature rise under simultaneous imaging and actuation (0.4
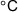
–0.6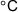) was comparable to that observed under actuation alone (0.1
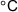
–0.4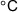), indicating that the RF contribution to total heating is small for the detuned catheter. For the axial coil, the difference between the two conditions was negligible ($<0.04^{\circ }C$). For the transverse coil, imaging added 0.5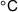 to the temperature rise; however, the total (0.6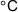) remained well below the FDA‐recommended 2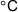 limit for the torso.^22^

The resulting model response is then given by $\Theta =H\left(s\right)\left(i^{2}R_{coil}\right)$ isolating the effects of Joule heating only. The unknown thermal resistance $R_{\theta }$ and heat capacitance $C_{\theta }$ are then determined by fitting the model H(s) to temperature data measured under Joule heating conditions only, where catheter coils are actuated without MR imaging. This fitting process minimizes the root mean square error (RMSE), $\varepsilon_{J}$, between the observed and model‐predicted temperatures:

(10) $$\left({\hat{R}}_{\theta },{\hat{C}}_{\theta }\right)=\underset{R_{\theta },C_{\theta }}{\mathrm{argmin}}\left(\sqrt{\frac{1}{N}\sum_{j=1}^{N}{\left(\Theta_{{meas}_{j}}-\Theta_{model{\left(R_{\theta },C_{\theta }\right)}_{j}}\right)}^{2}}\right),$$

where ${\hat{R}}_{\theta }$ and ${\hat{C}}_{\theta }$ are respectively estimated thermal resistance and heat capacitance.

${\hat{R}}_{\theta }$ and ${\hat{C}}_{\theta }$ represent effective thermal resistance and capacitance values fitted to observed data. These parameters are used to model thermal behavior at the system level and are not direct measurements of material properties or design targets.

### 2.4 Dithering

A dithering mechanism is introduced to modulate actuation currents by applying them in a 50% duty cycle at specific frequencies, resulting in mechanical oscillation or “shaking” of the catheter. A 10 Hz drive frequency that corresponds to a pulse duration of 50‐ms‐on, 50‐ms‐off is used. This frequency was selected to be compatible with real‐time MRI‐guided catheter tracking, where image acquisition and reconstruction operate at frame rates on the order of 10–20 frames per second.^38^, ^39^ The 50 ms on‐period ensures that the catheter tip maintains a stable deflection during each imaging frame, while the 50 ms off‐period provides the cooling interval. This pattern produces high‐frequency, low‐amplitude (submillimeter) oscillations at the catheter tip that enhance convective cooling by agitating the surrounding fluid. The oscillations are superimposed on the statically controlled tip position and do not introduce drift or displacement, thereby preserving the accuracy and stability required for precise targeting during RF ablation. The effect of the dithering on actuation behavior and target localization is further discussed in Ali et al.^4^

Forced thermal convection along the conducting wires from the dithering is expected to dissipate heat generated by the microcoils, thereby effectively decreasing the temperature of the prototype during actuation and reducing the risk of overheating.

### 2.5 Experimental setup

ISO/TS 10974^40^ provides a tiered framework for active implantable medical devices (AIMDs) but does not extend to partially implanted or external patient‐contacting devices. FDA guidance^41^ likewise notes that no standardized RF heating methods exist for such devices. Accordingly, a custom test protocol was developed to reflect the catheter's intended use. For comparison, standard‐compliant tests were also performed following ASTM F2182,^42^ with results in Figures S5 to S7 and Tables S5 and S6.

In order to evaluate the catheter prototype under MRI conditions, a custom phantom was constructed using a 4‐inch nominal Schedule 40 PVC pipe (inner diameter approximately 10.2 cm) approximately 173 cm (1.73 m) in length, holding 14 liters of water with a salt concentration of 1 gram per liter. The saline‐doped water phantom was used to enable real‐time actuation and imaging during RF heating tests, reflecting conditions relevant to cardiac ablation procedures.

The temperature of the coils was measured using Advanced Energy's Luxtron^®^ m924 OEM module.^43^ This fiber‐optic thermometer is capable of handling processes involving strong magnetic fields and high voltages, making it appropriate for measuring RF‐induced heating in the novel catheter prototype during actuation under MRI conditions. This measurement approach is consistent with fiber‐optic thermometry protocols used in prior RF heating evaluations of MRI catheter steering coils.^30^

Four fiber‐optic temperature probes were utilized, each positioned to monitor temperature at specific locations along the catheter prototype during each test. The probe placements are as follows:

1. **Probe One**: was positioned on the outer surface of the coil assembly to capture external heating and measures the temperature of the coils at the catheter tip where coil windings are most dense, leading to maximal Joule heating.
2. **Probe Two**: was routed along the internal leadwire within the catheter lumen, which was unsealed and exposed to the saline phantom. Tracks the temperature inside the lumen along the catheter shaft, drawn manually through the catheter during the tests to detect hot spots. The thin catheter tubing allows internal resistive heating to propagate outward, making this measurement relevant for thermal safety evaluation.
3. **Probe Three**: Monitors the temperature of the PCB (Figure 3b) connecting the catheter to the current‐driving amplifier, which rises considerably when the amplifier is active and serves as a timing reference as the thermal reading from Probe One is often diminished due to catheter dithering.
4. **Probe Four**: Acts as a control, extending beyond the catheter tip to detect ambient temperature fluctuations within the phantom.

**FIGURE 5:**
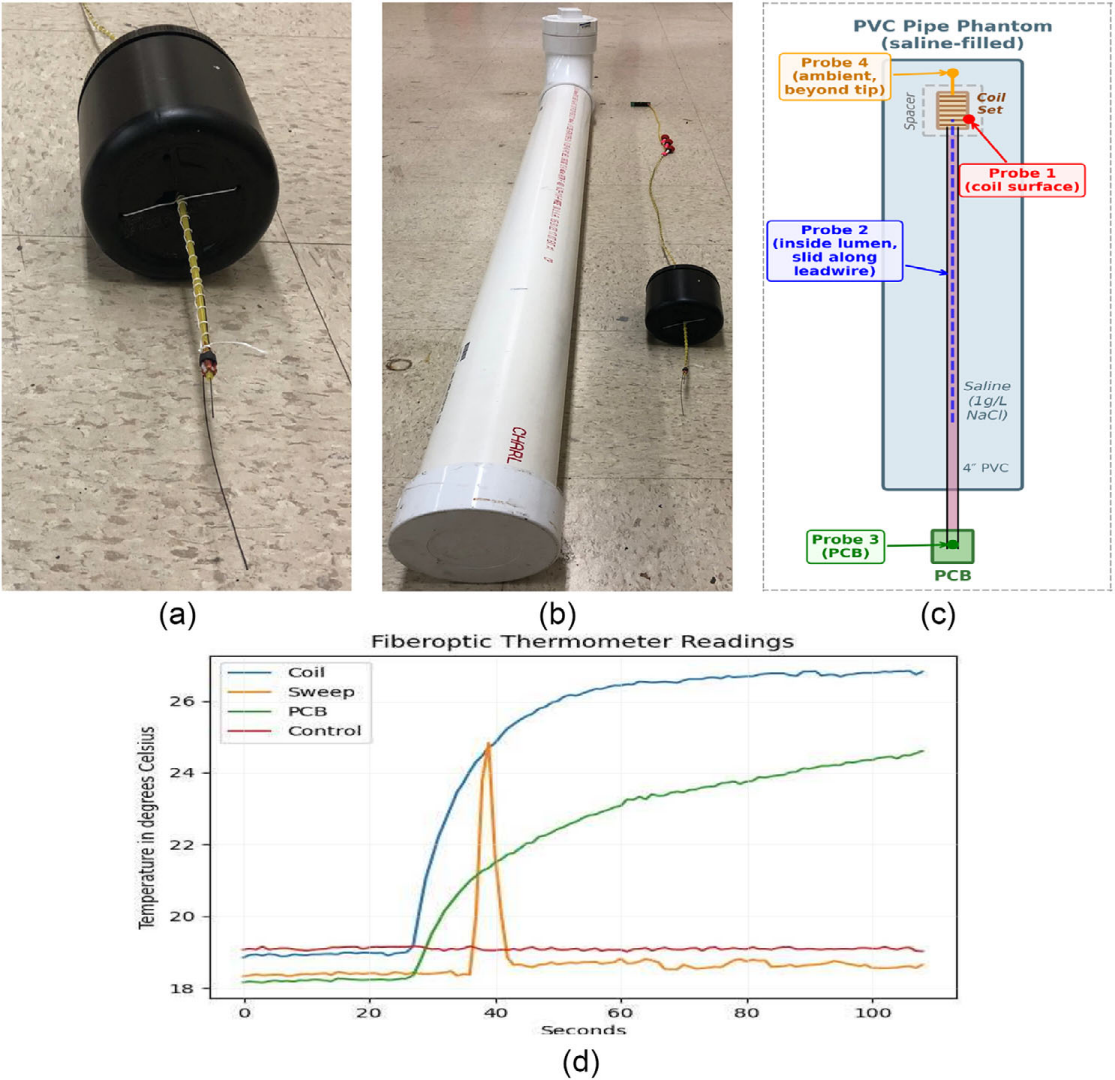
(a) Close‐up view of the catheter prototype with attached fiber‐optic thermometer probes and spacer. (b) The custom phantom and the catheter prototype placed next to it. (c) Schematic of the experimental setup showing the catheter inside the saline‐filled PVC pipe phantom with the four fiber‐optic temperature probe placements: Probe 1 on the coil surface, Probe 2 inside the catheter lumen (drawn along the leadwire to detect hot spots), Probe 3 on the PCB, and Probe 4 extending beyond the catheter tip as an ambient control. (d) Sample of raw thermal test data showing measurements from Probes 1–4, with a distinct spike on Probe 2 as it passes the coil assembly within the catheter lumen.

All probes were secured with fishing line using half‐hitch knots for stability, and a plastic spacer (Figure 5a) was placed around the catheter at the proximal end, where the coils sit, to keep it centered within the PVC pipe and prevent wall contact, without extending further down. The full catheter prototype equipped with fiber‐optic thermometer probes and an attached spacer for accurate temperature monitoring is shown alongside the phantom in Figure 5b. A schematic of the experimental setup and probe placements is shown in Figure 5c. An example of raw data collected during these tests is shown in Figure 5d, where a distinct spike in Probe Two temperature occurs as it is drawn past the coil assembly from within the catheter lumen.

In all the tests, the full catheter prototype (including all coil and leadwire segments) was immersed in the saline‐filled PVC pipe, with only the proximal PCB portion remaining outside the pipe. The catheter was inserted straight and oriented parallel to the scanner bore, which is aligned with the main magnetic field ($B_{0}$).

In the thermal tests that included MR imaging, a standard Balanced Steady‐State Free Precession (bSSFP) imaging sequence provided by the scanner vendor (Siemens 3T MAGNETOM Vida) was used to assess the heating characteristics of the design. The imaging parameters were as follows: repetition time (TR): 2.72 ms, echo time (TE): 1.19 ms, and flip angle (degrees): 50 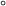 (Figure S4).

### 2.6 Statistical analysis

Temperature measurements were sampled at 1 Hz using fiber‐optic probes during each trial. For each experimental condition, the steady‐state region was identified as the interval over which the Probe 1 (coil surface) time series maintained a stable plateau following the initial thermal transient. The temperature rise was computed as:

(11) $$\Delta T=\left({\bar{P_{1}}}_{\mathrm{ss}}-{\bar{P_{1}}}_{\mathrm{bl}}\right)-\left({\bar{P_{4}}}_{\mathrm{ss}}-{\bar{P_{4}}}_{\mathrm{bl}}\right),$$

where ${\bar{P_{1}}}_{\mathrm{ss}}$ and ${\bar{P_{1}}}_{\mathrm{bl}}$ are the mean Probe 1 readings during the steady‐state and baseline (first 10 s) intervals, respectively, and $\bar{P_{4}}$ terms provide ambient drift correction from the control probe. Results are reported as mean ± standard deviation (SD).

Because consecutive temperature samples within a single trial are temporally correlated, the effective sample size $n_{\mathrm{eff}}$ was computed for each trial to account for autocorrelation:

(12) $$n_{\mathrm{eff}}=\frac{n}{1+2\sum_{k=1}^{K}\hat{\rho }\left(k\right)},$$

where n is the number of steady‐state samples, $\hat{\rho }\left(k\right)$ is the estimated autocorrelation at lag k, and the summation is truncated at the lag K where $|\hat{\rho }\left(k\right)|<0.05$. All confidence intervals, standard errors, and degrees of freedom use $n_{\mathrm{eff}}$ rather than n. For each trial, the steady‐state interval was taken as the plateau region following thermal equilibration, and n denotes the number of temperature samples within this interval; the resulting $n_{\mathrm{eff}}$ for each trial is reported with the corresponding results, and the reported confidence intervals therefore characterize within‐trial measurement variability.

Compliance with the FDA‐recommended temperature limit of 2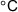 for the torso^22^ was assessed using a one‐sample, one‐sided t‐test ($H_{0}$: $\Delta T\geq 2^{\circ }C$, $H_{1}$: $\Delta T<2^{\circ }C$, α=0.05) with $n_{\mathrm{eff}}-1$ degrees of freedom. To assess whether MR imaging introduces additional heating in the axial‐coil configuration, where the design is expected to suppress RF coupling, a two one‐sided tests (TOST) equivalence procedure was used, since a non‐significant difference cannot by itself establish the absence of an effect.^44^, ^45^, ^46^ The equivalence margin was set to $\Delta =0.5^{\circ }C$, determined by the fiber‐optic thermometer accuracy and 25% of the FDA limit and therefore independent of the observed data. Equivalence was concluded by rejecting both one‐sided null hypotheses ($H_{0}^{L}$: $\mu_{d}\leq -\Delta$; $H_{0}^{U}$: $\mu_{d}\geq +\Delta$) at α=0.05, with degrees of freedom from the Welch–Satterthwaite approximation. For the transverse‐coil configuration, where a non‐zero RF contribution was anticipated, the imaging comparison was reported with a two‐sample Welch t‐test. The practical impact of dithering is quantified as the percentage reduction in ΔT relative to the corresponding DC condition.

## 3 RESULTS

### 3.1 RF response characterization

**FIGURE 6:**
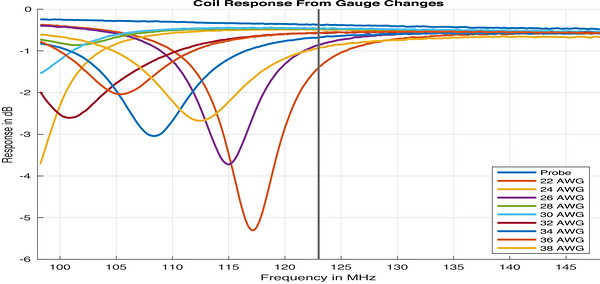
The reflection coefficients of various axial coil configurations are shown. The coupling probe response is given as a baseline for comparison.

The reflection coefficient $S_{11}$ for each axial coil prototype constructed with various wire gauges and configurations (Section 2.2.2) is shown in Figure 6. For all wire gauges, the self‐resonance peak appears below the 123 MHz Larmor frequency, with no clear trend observed between wire gauge and reflection coefficient. Several wire gauges proved unsuitable for use in a robotic catheter. Wires of 32 AWG or thicker reduced catheter flexibility, adding undesirable shape memory, while wires thinner than 36 AWG were fragile and challenging to strip or solder. A 33 AWG wire was chosen for the axial coils, thin enough to maintain an acceptable catheter width after winding the transverse coils, while staying within the allowable two‐layer limit (Table S2).

**FIGURE 7:**
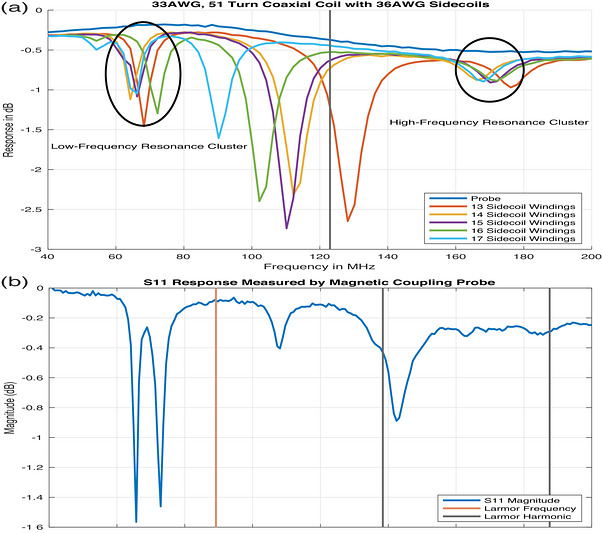
(a) Reflection coefficient of a coil set with various transverse coil configurations. The coupling probe response is shown as a baseline. (b) $S_{11}$ response of the finalized full‐size catheter prototype. No notable resonances were observed over the frequency range of interest. Sniffer probe measurements were obtained across multiple orientations; coupling strength varied, while resonance frequencies remained stable.

The RF response of the transverse coils is examined next, where a 36 AWG wire was deemed suitable for transverse coils due to physical dimension constraints. As with the axial coil, reducing the transverse coil turn count diminishes the catheter's actuation capability, with turn numbers constrained by bobbin dimensions. The reflection coefficients $S_{11}$ for different coil sets with a 33 AWG, 51‐turn axial coil and 36 AWG side coils with various winding turns are shown in Figure 7a. Three distinct peaks were observed: a low‐frequency peak below 80 MHz, a middle peak near the 123 MHz Larmor frequency with large variation, and a high‐frequency peak above 160 MHz. Increasing transverse coil turns shifts the central resonant peak away from the Larmor frequency, with minimal effect on the low‐ and high‐frequency resonances. The reflection coefficient $S_{11}$ of the final catheter prototype is shown in Figure 7b, with no notable resonances observed across the frequency range. Sniffer probe measurements were obtained by sweeping across all coil orientations. While coupling strength varied with direction, resonance frequencies remained stable and were used for design evaluation. The final catheter assembly is shown in Figure S3.

### 3.2 Thermal response analysis

#### 3.2.1 Joule heating analysis

The thermal tests for Joule heating analysis were conducted by first actuating the axial coil while keeping the transverse coils off, followed by the activation of a single transverse coil while the axial coil remained off. Two actuation currents were tested: direct current (DC) and a 50% duty cycle with a 50‐ms‐on, 50‐ms‐off pulse.^5^ MRI imaging was not performed, keeping the RF transmitter powered off, thereby eliminating any 123 MHz environmental signal. This setup ensured that the thermal tests isolated the effects of Joule heating generated by the amplifier alone, free from MRI‐induced RF interference. The amplifier driving the catheter had a maximum output of 1 A.^47^ All trials were conducted at this maximum current to evaluate Joule heating under worst‐case conditions.

**FIGURE 8:**
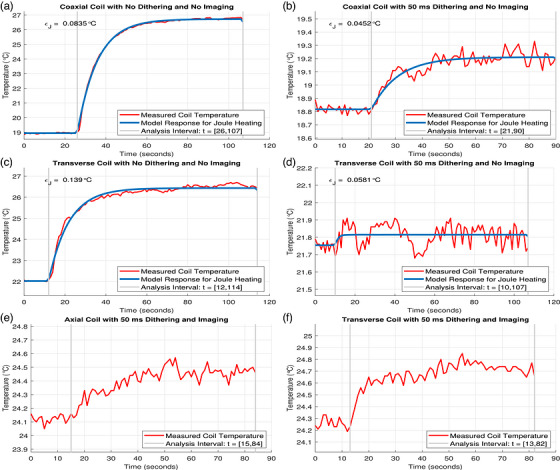
The temperature changes along the coils due to Joule and RF heating are presented. PCB heating serves as an indicator to confirm current flow through the coils and is used to determine the analysis interval, as shown in the figures. (a) Joule Axial Coil DC. (b) Joule Axial Coil 50 ms‐on, 50 ms‐off. (c) Joule Transverse Coil DC. (d) Joule Transverse Coil 50 ms‐on, 50 ms‐off. (e) RF Axial Coil 50 ms‐on, 50 ms‐off. (f) RF Transverse Coil 50 ms‐on, 50 ms‐off.

The results from these four trials are presented in Figure 8a–d, where the RMSE $\varepsilon_{J}$ (10), between the measured temperatures and model response is shown for each trial.

In the axial coil experiments, the DC trial (Figure 8a) produced a steady‐state temperature rise of 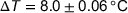 ($n_{\mathrm{eff}}=13$, 95% CI: [7.96, 8.04]), while the 50 ms on/off dithered configuration (Figure 8b) reduced this to 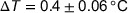 ($n_{\mathrm{eff}}=28$, 95% CI: [0.38, 0.42]), a 95% reduction. In the transverse coil, the DC trial (Figure 8c) produced 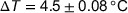 ($n_{\mathrm{eff}}=10$, 95% CI: [4.44, 4.56]), whereas the dithered configuration (Figure 8d) reduced the temperature rise to 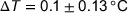 ($n_{\mathrm{eff}}=16$, 95% CI: [0.03, 0.17]), a 98% reduction.

Under both dithered conditions, the measured ΔT was below 2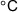 with the upper bound of each 95% confidence interval remaining below 1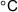 (one‐sample, one‐sided t‐test, p<0.001; Table 2).

**TABLE 2:** Summary of steady‐state temperature rise under each experimental condition.

Condition	ΔT ( 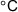 ) ↓	SD ( 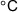 )	$n_{\mathrm{eff}}$	95% CI ( 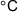 )	Reduction
Axial DC, No Imaging	8.0	0.06	13	[7.96, 8.04]	—
Axial 50ms, No Imaging	0.4^*^	0.06	28	[0.38, 0.42]	95%
Transverse DC, No Imaging	4.5	0.08	10	[4.44, 4.56]	—
Transverse 50ms, No Imaging	0.1^*^	0.13	16	[0.03, 0.17]	98%
Axial 50ms, Imaging	0.4^*^	0.06	29	[0.38, 0.42]	—
Transverse 50ms, Imaging	0.6^*^	0.07	12	[0.56, 0.64]	—

*Note*: ↓ indicates that a lower value is favorable. Reduction is relative to the corresponding DC no‐imaging condition for the same coil type.

^*^ p<0.001 (one‐sample, one‐sided t‐test against 2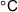 FDA limit).

#### 3.2.2 RF heating analysis

Additional tests were conducted to observe the effect of RF‐induced heating. Joule heating experiments in Section 3.2.1 demonstrated the role of dithering in enhancing heat dissipation during actuation. Consequently, the thermal tests for RF heating analysis were conducted only with 50% duty cycle actuation patterns employed in Joule heating experiments but with simultaneous MR imaging, which was not performed in the Joule heating tests. This distinction enabled analyzing the heating effects from MRI‐induced RF signals. The results for the two dithering‐based actuation patterns are presented in Figure 8e,f where the measured temperatures are shown for each trial.

In the axial coil experiments with simultaneous imaging, the 50 ms on/off pulsed trial (Figure 8e) resulted in a temperature rise of 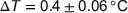 ($n_{\mathrm{eff}}=29$, 95% CI: [0.38, 0.42]). The imaging‐on and imaging‐off conditions were statistically equivalent within the equivalence margin of $\Delta =0.5^{\circ }C$ (TOST on 54.9 degrees of freedom, both one‐sided tests rejected at p<0.001; 90% CI on the mean difference 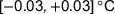).

In the transverse coil experiments, the imaging trial (Figure 8f) produced 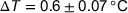 ($n_{\mathrm{eff}}=12$, 95% CI: [0.56, 0.64]), higher in absolute terms than the corresponding no‐imaging condition (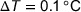, Welch t‐test, t(24.0)=13.06, p<0.001). The resulting temperature rise remained within the FDA limit of 2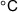.

#### 3.2.3 Detection of hot spots along the catheter leadwire

**FIGURE 9:**
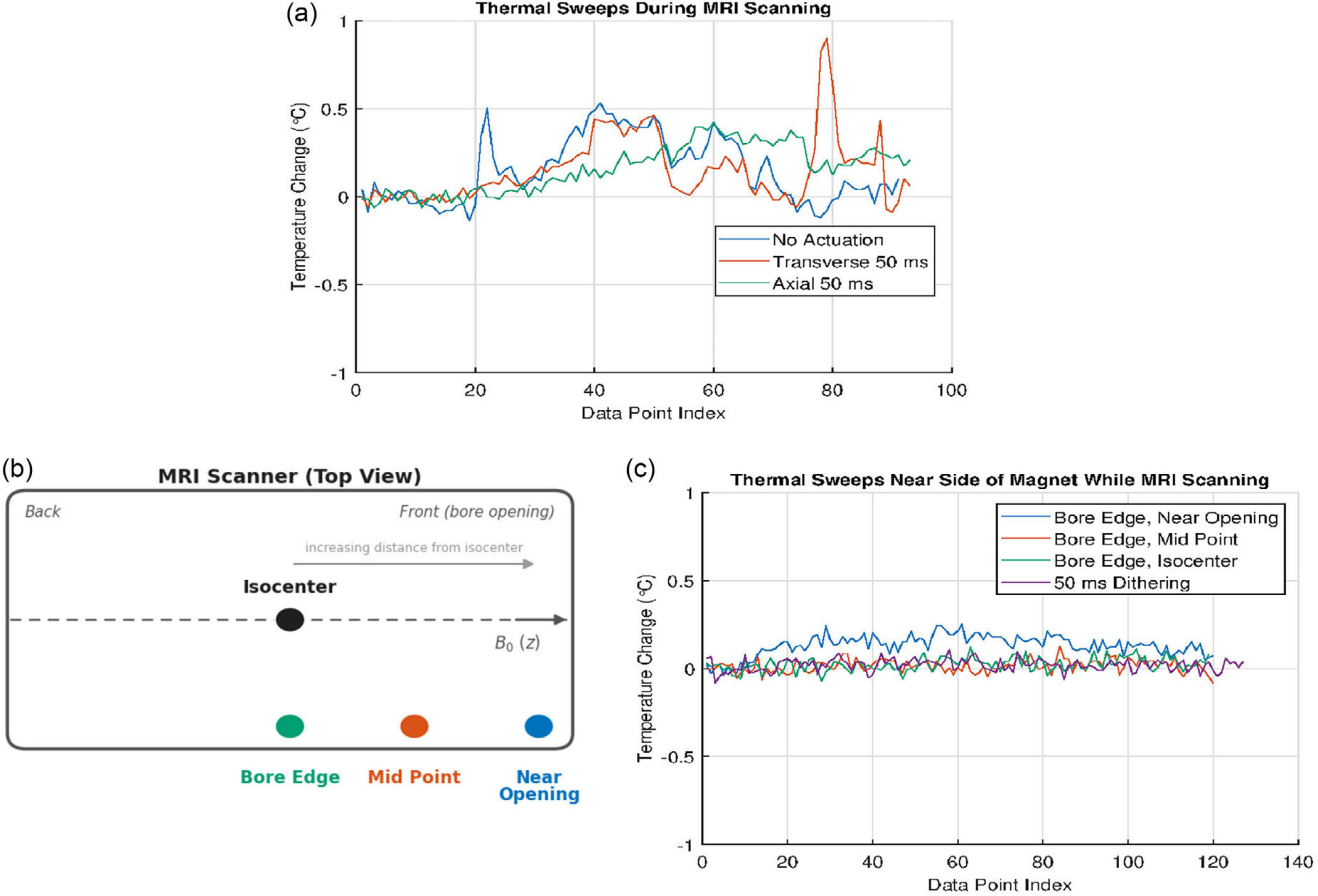
(a) Temperature changes recorded during the thermal sweeps are shown. No actuation case is provided as a baseline. (b) Measurement locations used to examine the spatial dependency of RF heating are shown. (c) Coil temperatures at various positions away from the isocenter of the MRI scanner.

In each RF heating test (Section 3.2.2), a temperature probe was moved along the catheter leadwires to detect hot spots that can form due to RF‐induced resonance and localized heating due to standing wave effects. These thermal sweeps can be seen in Figure 9a.

The temperature probe exhibited a variation of $+0.9^{\circ }C$ and $-0.1^{\circ }C$ relative to its baseline. No location along the catheter demonstrated a consistent temperature increase, and there were no temperature changes above 1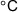.

#### 3.2.4 RF thermal assessment away from the isocenter

The uniform magnetic field of the MRI scanner is supplied by a large solenoid of finite length, which produces an approximately homogeneous field near the imaging volume. However, near the ends of the solenoid, deviations from field homogeneity occur as the previously parallel and closely spaced magnetic field lines begin to diverge.^37^

Additional tests were carried out to assess spatial resonance behavior away from the isocenter of the MRI. Four measurements were taken using a custom MRI phantom positioned near the edge of the MRI bore and a Siemens Loaded Body Phantom placed at the center to simulate realistic loading conditions. This setup ensured that the RF transmitter emitted a strong transmit field comparable to that observed with a human subject, effectively mimicking real‐world application conditions for future use of the catheter prototype.

The measurement locations are shown in Figure 9b. The first three were taken without catheter actuation; the edge farthest from the isocenter, the boundary at the opening of the magnet bore, and the midpoint between the edge and the opening. The remaining test was conducted with the catheter positioned at the opening of the magnet's bore, representing the boundary condition of the nonuniform magnetic field. During these tests, the axial coil was actuated using the 50 ms dithering pattern. These four test cases can be seen in Figure 9c.

It can be observed that the temperature changes exhibit minimal variance throughout the bore, and the behavior of the catheter remains consistent across varying magnetic field strengths, providing evidence of reliable thermal performance across spatial variations within the bore.

## 4 DISCUSSION

The results demonstrate that both RF‐induced and resistive heating can be effectively mitigated in the presented full‐length, magnetically actuated MRI‐guided catheter through the combined use of distributed capacitors, coil detuning, and a dithering‐based convection strategy. Under simultaneous imaging and 1 A dithered actuation, the maximum temperature rise was 0.6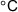 at the transverse coil (Figure 8f) and 0.4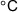 at the axial coil (Figure 8e), both well within the FDA‐recommended limit of 2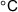 for the torso.^22^

Under all dithered actuation conditions, the upper bound of the autocorrelation‐corrected 95% confidence interval for the temperature rise remained below 1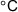 (Table 2), providing a safety margin of at least 1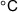 relative to the FDA limit (one‐sample, one‐sided t‐test, p<0.001 for all conditions). Dithering reduced the steady‐state temperature rise by 95% for the axial coil (from 8.0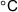 to 0.4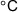, Figure 8a,b) and by 98% for the transverse coil (from 4.5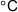 to 0.1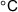, Figure 8c,d), demonstrating that duty‐cycled actuation is effective at suppressing resistive heating in both coil geometries.

Distributed capacitors integrated along the conductive paths disrupted standing‐wave formation and suppressed resonance that could otherwise lead to localized heating. The detuned coil geometry further minimized coupling with the transmit RF field not only at the 123 MHz Larmor frequency but also across its relevant harmonics, providing broadband suppression (Figure 7b). Thermal sweeps along the catheter leadwires confirmed uniform temperature behavior, with no location exhibiting a temperature change above 1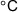 (Figure 9a).

The 50% duty‐cycle actuation mode reduced resistive heating by introducing short cooling intervals between applied currents. Under DC actuation, the axial coil produced a temperature increase of 8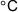 (Figure 8a), whereas the 50 ms on/off dithered pattern reduced this to 0.4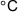 (Figure 8b). A similar reduction was observed in the transverse coil, from 4.5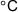 under DC to 0.1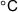 with dithering (Figure 8c,d).

The dithering mechanism produces submillimeter oscillations at the catheter tip at 10 Hz. These oscillations do not affect the time‐averaged tip position and do not introduce drift or instability during navigation. Catheter vibration as a means of enhancing convective electrode cooling has been independently demonstrated by Yu et al.,^48^ and a detailed characterization of dithering effects on actuation performance and target localization for the present catheter system is presented in Ali et al.^4^

For the axial coil, the imaging‐on and imaging‐off conditions were statistically equivalent within the 0.5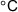 equivalence margin (TOST, both one‐sided tests rejected at p<0.001; Table 2), providing positive evidence that any imaging‐induced contribution is smaller than the 0.5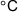 measurement‐accuracy margin rather than an effect left undetected by limited statistical power, consistent with effective detuning of the axial coil. For the transverse coil, a 0.5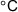 increase in absolute terms was observed with imaging (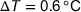 vs. 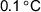, Welch t‐test, t(24.0)=13.06, p<0.001), consistent with a minor RF contribution; however, the resulting temperature rise remained well below the FDA limit of 2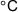. These findings are consistent with prior work by Settecase et al.,^30^ who reported that resistive heating from actuation currents dominates over RF‐induced heating in MRI catheter steering coils.

RF‐induced heating of the assembled catheter was characterized by direct fiber‐optic temperature measurement during MRI RF exposure; the maximum temperature rise was 0.6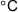, within the 2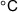 FDA limit.^22^ At the 123 MHz Larmor frequency, the wavelength in the 1 g/L saline test medium is approximately 27 cm, and the catheter leadwires extend approximately 1.5 m. The distributed capacitors shift leadwire resonances away from the Larmor frequency, and the measured reflection coefficient ($S_{11}$) of the finalized full‐size catheter prototype shows no resonance near 123 MHz (Figure 7b).

Spatial mapping across multiple bore positions showed consistent thermal performance with no position‐dependent resonances or hot spots (Figure 9b,c), indicating that the distributed circuit and coil design are robust to variations in field distribution and loading conditions inside the scanner. The small probe‐to‐probe variation observed during the hot‐spot sweeps ($+0.9^{\circ }C$ to $-0.1^{\circ }C$) is attributable to differences in the temperature distribution along the phantom and to minor RF heating, and did not correspond to any consistent localized temperature increase.

Overall, the presented catheter design effectively mitigates both RF‐induced and resistive heating across the entire device length. The combination of distributed capacitors, coil detuning, and duty‐cycled current delivery maintains the thermal safety of the magnetically actuated, MRI‐guided robotic catheter.

### 4.1 Limitations

The present study has several limitations. When the catheter tip is in contact with the vessel wall, fluid flow around the tip is restricted, which would reduce the convective cooling provided by dithering. However, wall contact typically corresponds to ablation delivery, where localized heating at the tissue interface is the intended therapeutic effect. The thermal results in this study were obtained with the catheter in a free‐fluid environment, representing the operating condition where dithering‐based cooling is most relevant.

The use of saline rather than blood as the phantom medium is a limitation of this study. Blood has higher viscosity than saline (∼4.0 mPa·s vs. ∼0.7 mPa·s at 37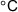 ^48^) and different thermal properties, which may reduce the effectiveness of dithering‐based forced convection in vivo. However, the maximum temperature rise observed (0.6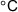) was well below the FDA‐recommended limit of 2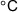 for the torso,^22^ providing a safety margin that accommodates the expected differences in convective heat transfer between the two media. The use of saline as a blood‐equivalent medium is consistent with established in vitro catheter ablation testing protocols.^49^, ^50^ Validation with blood‐mimicking fluids remains a direction for future work.

The catheter design is tailored to a 3T field strength, and translation to a different $B_{0}$ would require re‐tuning the coil configuration and capacitor placement for the corresponding Larmor frequency. However, the design methodology is directly transferable, and field‐specific device‐scanner pairing is standard practice for regulatory approval of MRI‐compatible devices.

This study evaluates a single catheter prototype, and the reported statistics characterize measurement variability within individual trials. Because RF‐induced heating through antenna and Joule effects is sensitive to microscopic manufacturing tolerances and micro‐coil alignment, results from one prototype may not capture geometric variability across devices. Temporal autocorrelation in the fiber‐optic probe data was accounted for through effective sample sizes (Equation 12), so the reported confidence intervals reflect within‐trial precision rather than cross‐session stability or setup‐repositioning reproducibility, which remain unquantified. The safety margin between the observed maximum temperature rise (0.6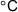) and the FDA limit (2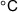) provides confidence that the design would remain compliant under moderate increases in variability, and formal characterization of inter‐device, cross‐session, and setup‐repositioning reproducibility across multiple prototypes is planned as part of future pre‐clinical validation.

This study specifically focused on RF‐induced and resistive heating mitigation and did not include magnetic torque characterization per ASTM F2213‐17, which falls outside the scope of this thermal safety investigation.

## 5 CONCLUSION

This study evaluated the thermal safety of a magnetically actuated MRI catheter incorporating distributed capacitors, coil detuning, and duty‐cycled dithering. Under simultaneous imaging and 1 A actuation with a 50% duty cycle (10 Hz, 50 ms bursts), the maximum observed temperature increase was less than 1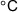, remaining within the FDA‐recommended limit of 2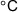. No localized heating was observed along the catheter leadwires, and thermal performance remained consistent across all tested bore positions. These results indicate that the combined mitigation strategies maintained thermal safety under the tested conditions; however, further testing across a broader range of configurations is needed to confirm general compliance with safety standards.

Future work will include in vivo validation, expanded testing across additional scanner configurations and operating parameters, and incorporation of electrical safety features aligned with IEC 60601‐1 requirements.

## CONFLICT OF INTEREST STATEMENT

The authors declare no conflicts of interest.

## FUNDING INFORMATION

This work was supported in part by the National Institutes of Health, under grants R01 HL153034 and R01 HL163991, and the National Science Foundation, under grants CISE IIS‐1563805 and ENG IIP‐1700839.

## ETHICS STATEMENT

This study did not involve human participants or animal subjects. All experiments were conducted using phantoms, and therefore no ethical approval was required.

## Supporting information



Supporting Information

## Data Availability

The data that support the findings of this study are not publicly available.

## Footnotes

1 3T Siemens MAGNETOM Vida

2 Agilent E8362B PNA Series Network Analyzer

3 Vishay Vitremon VJ0603D471KCXAJ 0603

4 Venkel Ltd. C0805Y5V250

5 A corresponding table showing the excitation parameters is provided in Table S4.
