# Supplementary material for: Magnetic resonance imaging safety‐driven, heat‐mitigating design for a magnetic resonance imaging‐guided, magnetically actuated robotic catheter
Source: Med Phys. 2026 Aug 10;53(8):e70630. doi: 10.1002/mp.70630 (PMC13455776; doi:10.1002/mp.70630)
Supplement: Supplementary file 1 [file MP-53-0-s001.pdf]

Magnetic Resonance Imaging Safety-Driven, Heat-Mitigating Design for a Magnetic Resonance Imaging-Guided, Magnetically Actuated Robotic Catheter

Supporting Information

Variables	Symbols (units)
Wire gauge of axial coil	w_{g_a} (AWG)
Wire gauge of side coil	w_{g_s} (AWG)
Winding number of axial coil	N_a
Winding number of transverse coils	N_s
Number of axial coil layers	n_l

Supporting Information Table S-1. Coil set configuration variables are shown. The wire gauge determines the wire thickness, affecting the number of winding turns, coil diameter, and current carrying capacity. The number of layers impacts the number of winding turns and coil diameter. The number of winding turns influences coil length, coil inductance, and the strength of the generated magnetic torque.

AWG	Wire Diameter (mm)	Number of Layers	Ampacity at 60°C (A)
22	0.644	1	3
24	0.511	1	2.1
26	0.405	1	1.3
28	0.321	1	0.83
30	0.255	2	0.52
32	0.202	2	0.32
34	0.160	3	0.18
36	0.127	4	0.11
38	0.101	5	0.07

Supporting Information Table S-2. The wire gauges that are considered for the coil design with the corresponding wire diameter, number of layers, and ampacity values.

AWG	Coaxial Coil Inductance (μH)
22	7.6
24	8.9
26	12.8
28	32.3
30	39.6
32	71.9
34	86.4
36	140.8
38	186.4

Supporting Information Table S-3. The wire gauges and the corresponding inductance values for the coaxial coil.

Test	Excitation	Duty Cycle / Frequency	Burst Duration	Total Duration
1	1 A DC	100% (DC)	–	100 s
2	1 A Pulsed	50% (10 Hz)	50 ms ON / 50 ms OFF	100 s

Supporting Information Table S-4. Summary of test excitation parameters. For the duty cycle test, a 1 A square-wave signal was applied in an ON–OFF–ON–OFF pattern with 50% duty cycle.

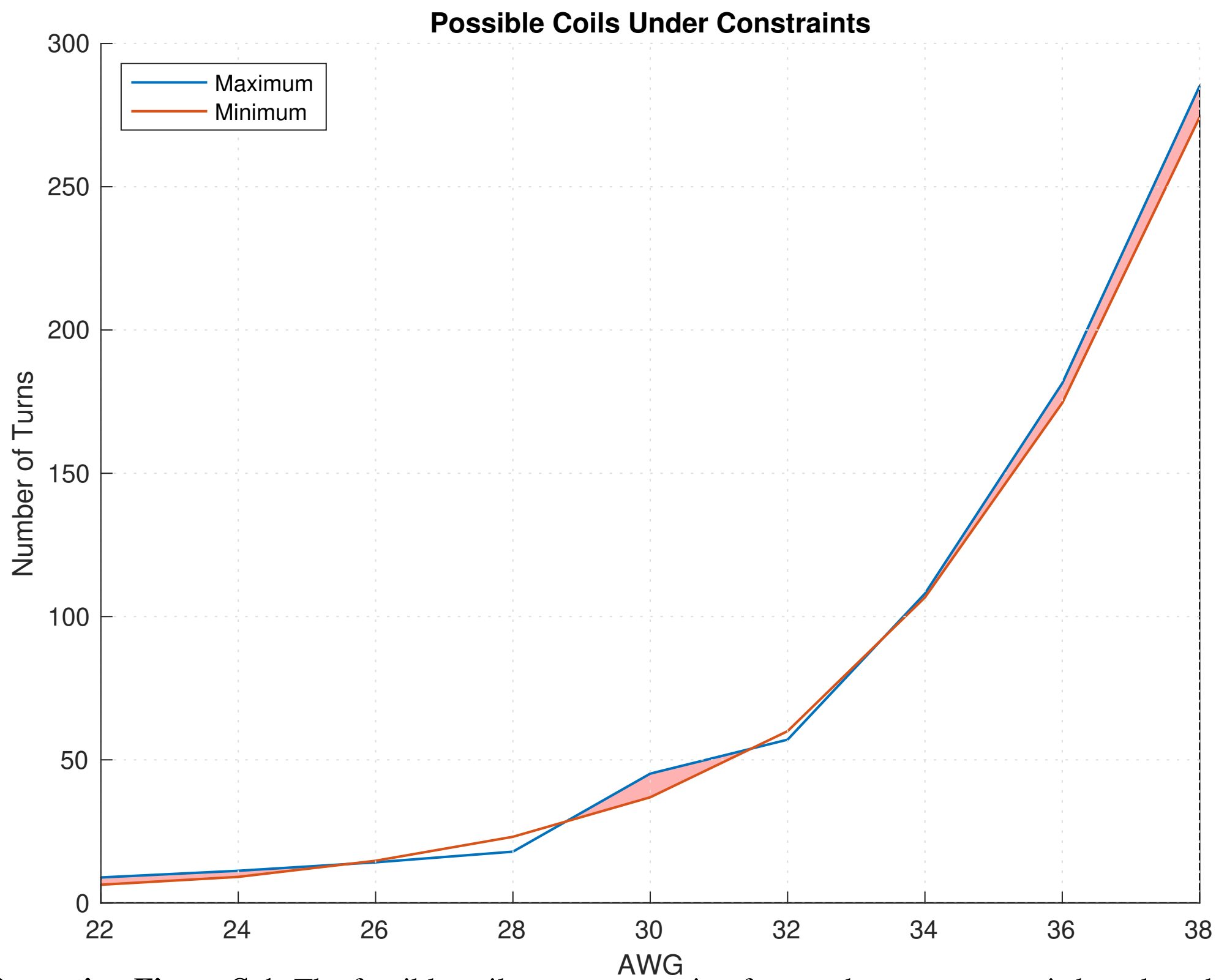

Supporting Information Figure S-1. The feasible coil turn range region for a catheter prototype is based on the maximum and minimum turn counts required to produce a functional axial coil. The shaded area represents this range, converging to a single optimal coil turn value for each wire gauge.

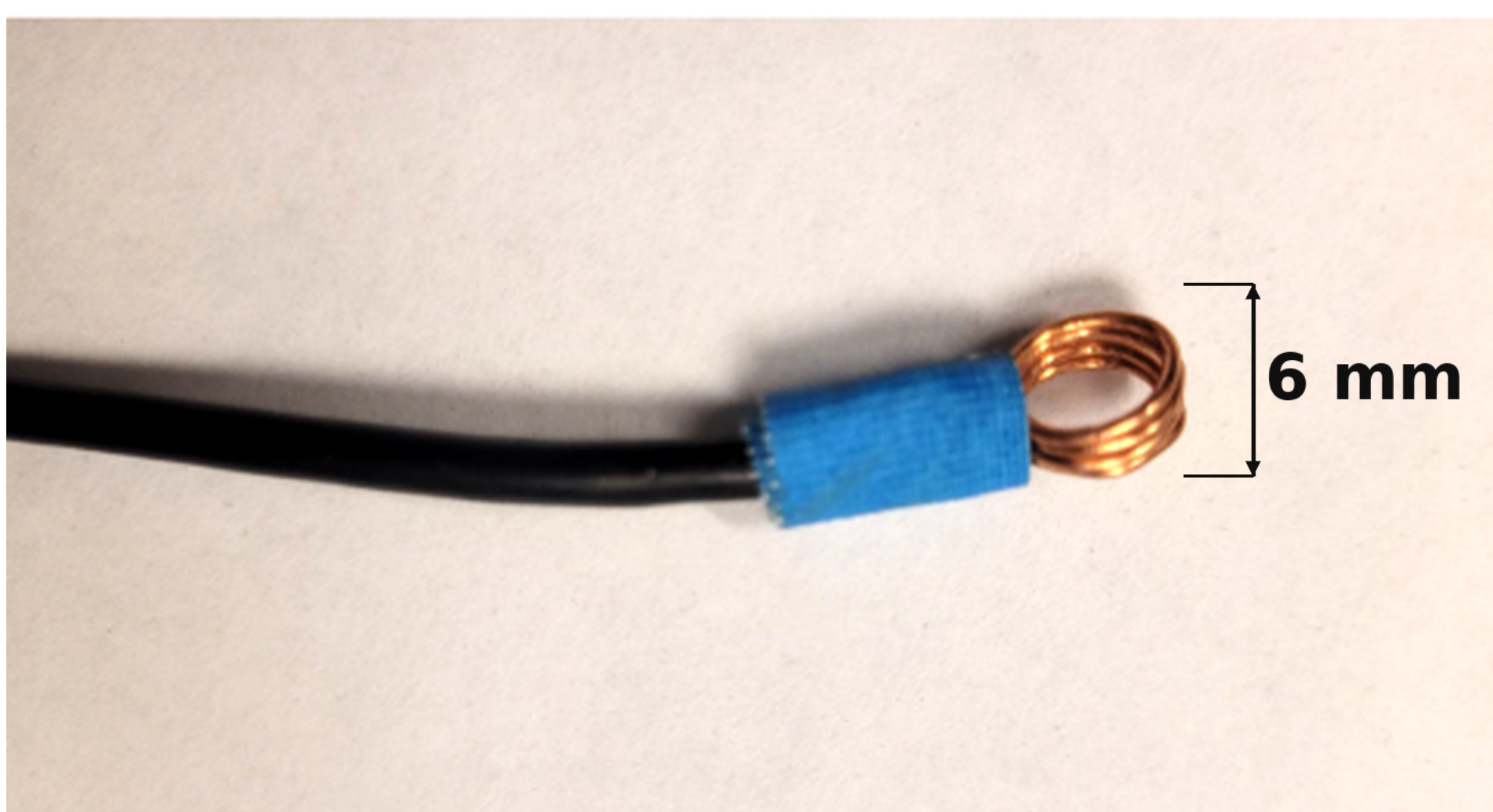

Supporting Information Figure S-2. The coupling probe, a 4-turn loop approximately 6 mm in diameter, is used to measure the reflection coefficient S_{11} of coils using a network analyzer.

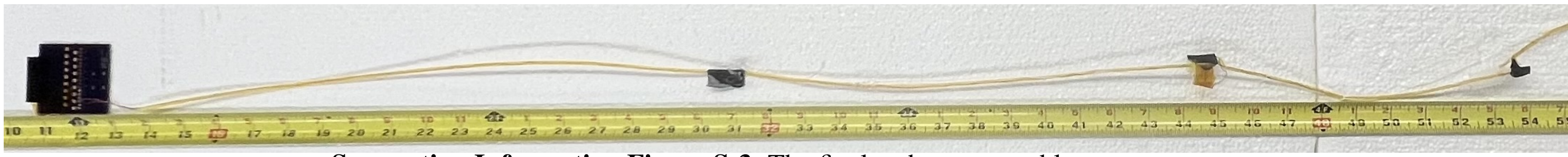

Supporting Information Figure S-3. The final catheter assembly.

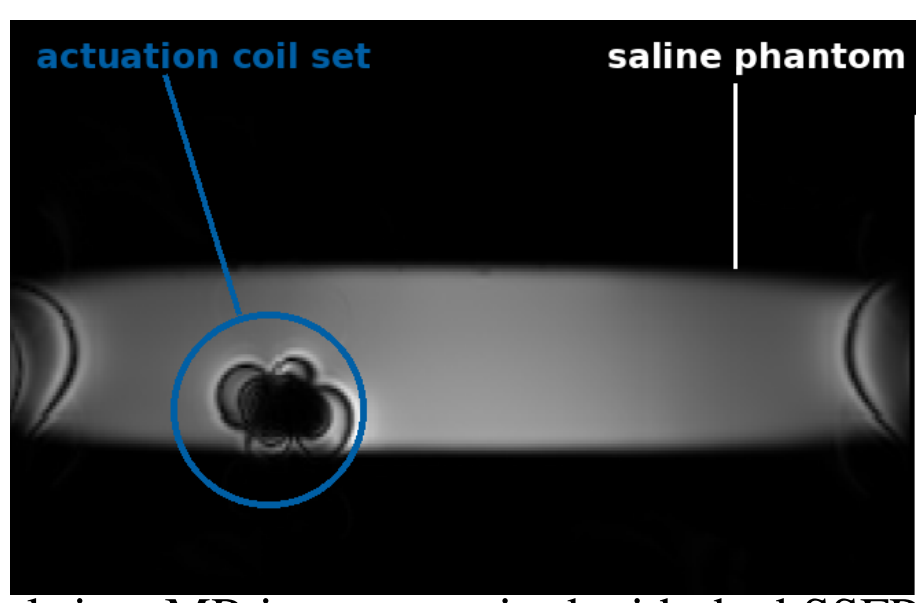

Supporting Information Figure S-4. Real-time MR image acquired with the bSSFP sequence during catheter operation in the saline phantom. The circled region indicates the actuation coil set.

ASTM F2182 Test

Methods. RF heating was assessed according to ASTM F2182 using a PAA gel–saline phantom in a PET container (54.7 cm × 38 cm × 15 cm)[1]. The gel was prepared by dissolving 151 g NaCl (S9888, Sigma-Aldrich, USA) and 20 g poly(acrylic acid) partial sodium salt (436364, Sigma-Aldrich, USA) in 4 gal (≈15.1 L) distilled water[1]. The catheter was suspended 2.6 cm above the phantom floor using two 3D-printed PLA holders, positioned near the side wall with ≥ 2 cm clearance from all boundaries. Although the ASTM F2182[1] example illustrates phantom dimensions of 65 cm × 42 cm × 9 cm, alternative geometries are permitted under Note 1 of the standard. The phantom used here provided an equivalent loading volume and maintained all required clearances.

Three fluoroptic probes (STB PROBE, LumaSense/Advanced Energy) were placed: (i) adjacent to the actuation coils, (ii) along the shaft near the proximal leadwire (both ≈3 cm from the implant holders), and (iii) a reference probe 30 cm away (Figs. S4–S5). Temperatures were recorded for 15 min at 1 Hz using a Luxtron m924 fluoroptic thermometer. A separate 15-min control run without the catheter was acquired at the same probe locations to characterize the background heating[1].

Probe positions were chosen in accordance with preliminary tests performed in the custom RF heating method developed in this work, identifying regions of expected maximum heating. The reference probe tracked ambient phantom heating. Boundary and holder effects were minimized by maintaining ≥2 cm clearance to phantom walls and spacing the holders more than twice their diameter away from probes; probe placement was verified before and after each run[1]. The room temperature was stable at 21.4 °C for 15 min prior to RF exposure and 21.1 °C for 15 min afterward. Heating was assessed under imaging conditions using a vendor-supplied bSSFP sequence (TR: 2.72 ms, TE: 1.19 ms, Flip Angle: 49 degrees).

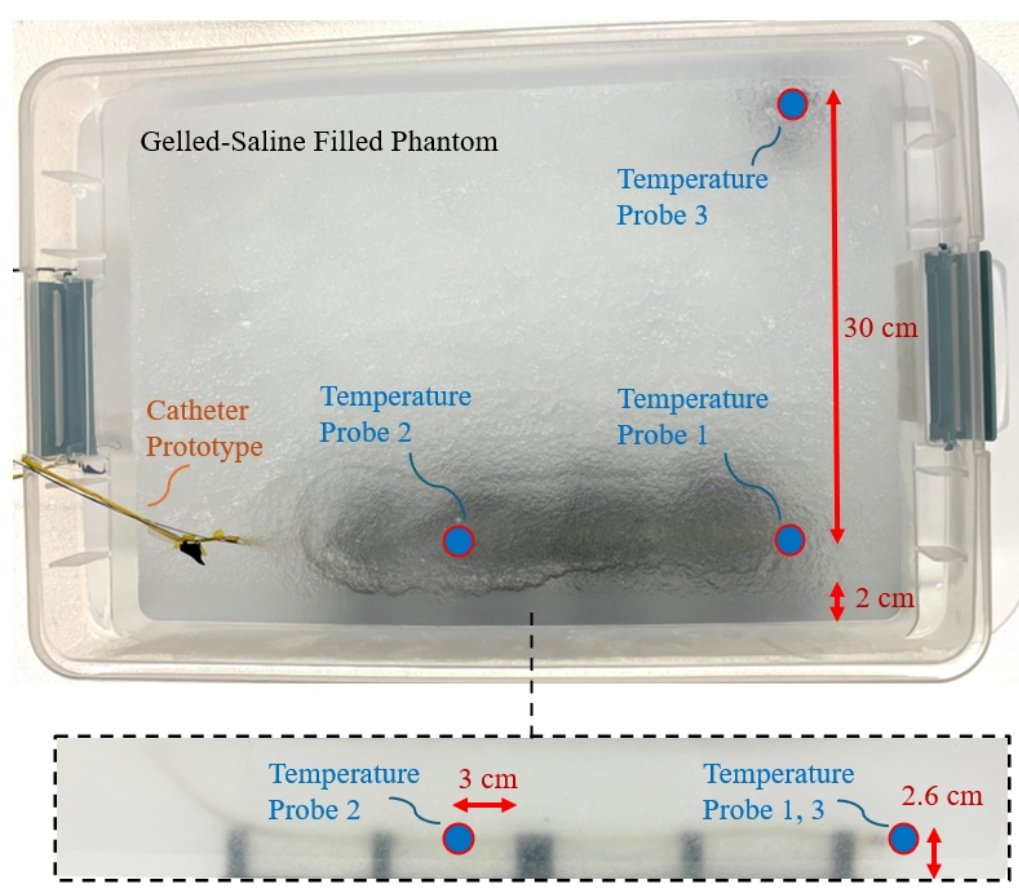

Supporting Information Figure S-5. ASTM F2182 experimental setup. The prepared phantom, along with the catheter and temperature probes are shown.

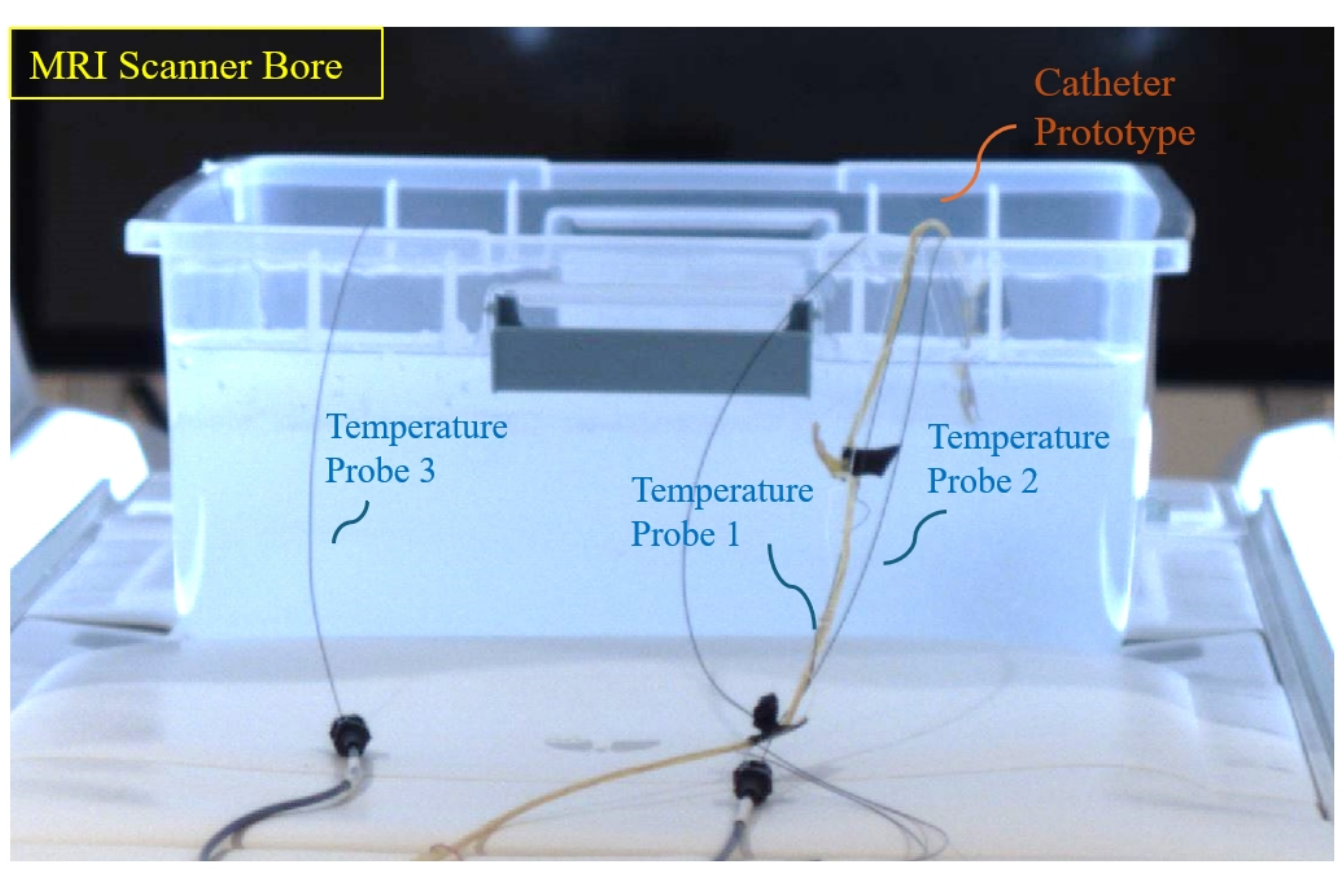

Supporting Information Figure S-6. The experimental setup inside the MRI scanner bore.

Results. Fig. S-7 shows unscaled temperature–time plots (catheter present: solid; no catheter: dashed). Maximum ΔT values over the 0–15 min exposure are given in Table S-5. Scaled values based on local background SAR are provided in Table S-6. Local background SAR was estimated from the no-catheter data using linear fits of dT/dt over 5–10 min and $c = 4150$ J/(kg °C)[1]. The proximal leadwire probe had a negligible background rate of temperature change; therefore, its scaled value is not reported (see Table S-6 note), while its unscaled response is included in Table S-5 and Fig. S-7.

The calculated local SAR values were 1.08 W/kg at the reference and 0.40 W/kg at the coil region. Corresponding scaled maximum temperature rises at SAR = 1 W/kg were 0.67°C at the reference and 1.05°C at the coil.

Measurement uncertainty. The Luxtron m924 datasheet[2] specifies probe precision of 0.01°C, accuracy within ±0.5°C, and temporal resolution <1 s. These specifications comply with the ASTM F2182 requirement that probes have precision ≤0.1°C, accuracy ≤0.5°C, and temporal resolution ≤2 s[1].

Conclusion. The maximum scaled temperature rises were below 1°C per W/kg at the actuation coils. These results suggest that, under the tested conditions, the catheter prototype does not exhibit excessive RF-induced heating and is within the expected safety limits defined by ASTM F2182.

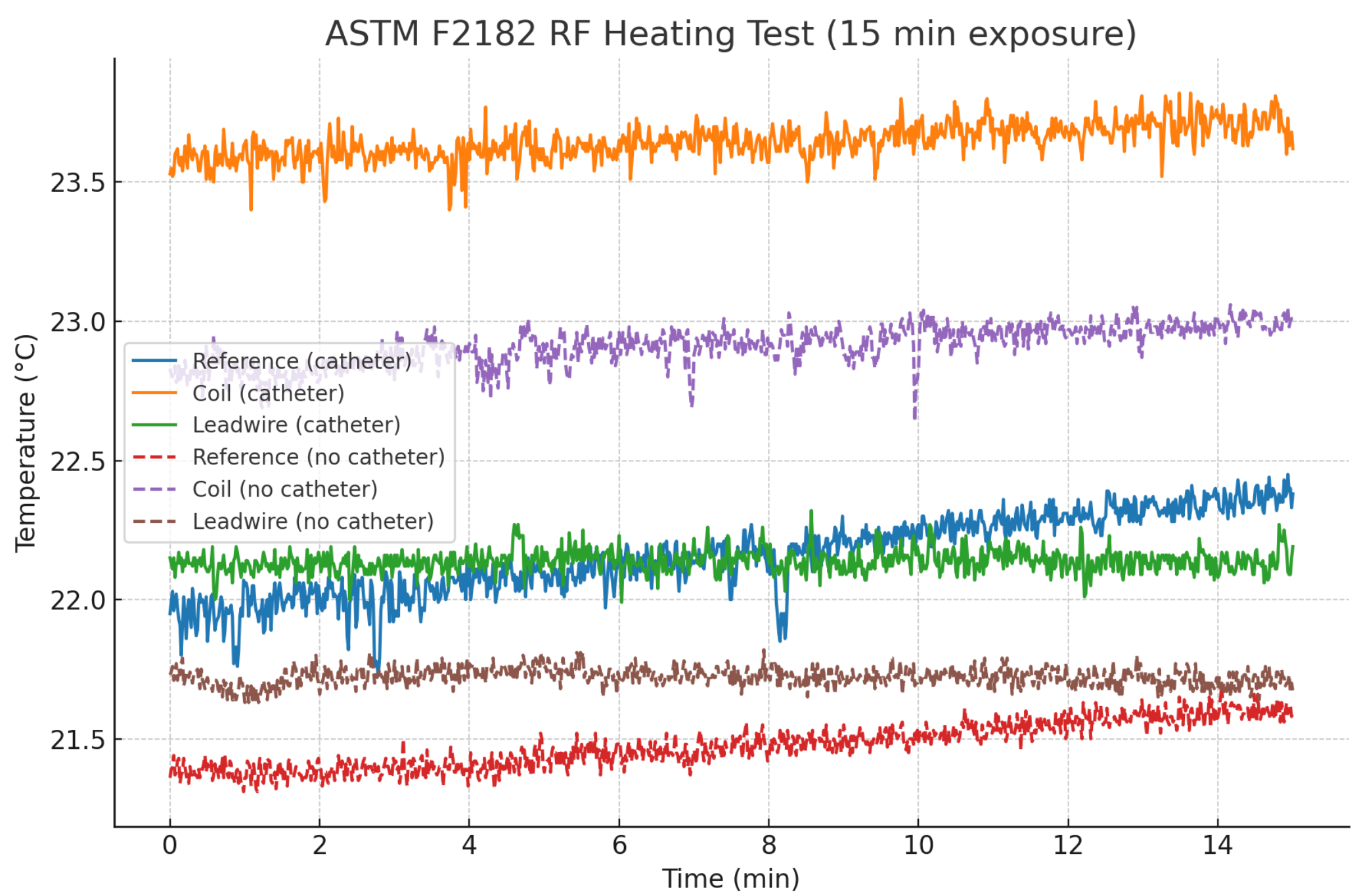

Supporting Information Figure S-7. ASTM F2182 RF Heating Test (15 min exposure). Temperature vs time at the reference, coil, and leadwire probes; catheter (solid) and no catheter (dashed).

Supporting Information Table S-5. Maximum unscaled temperature rise (ΔT, °C) over 0–15 min. ↓ = lower is favorable for safety.

Probe location	ΔT [°C] with catheter (↓)	ΔT [°C] no catheter (background)
Reference	0.72	0.38
Coil region	0.42	0.42
Proximal leadwire	0.33	0.19

Supporting Information Table S-6. Scaled results per ASTM F2182 using local background SAR from the no-implant run (5–10 min window). $c = 4150$ J/(kg·°C). ↓ = lower is favorable for safety.

Location	ΔT (catheter) [°C]	SAR_{local} [W/kg]	ΔT @ SAR=1 [°C] (↓)	°C/((W/kg)·min) (↓)
Reference	0.72	1.08	0.67	0.044
Coil	0.42	0.40	1.05	0.070

Note: Leadwire scaled values are not reported due to a near-zero background slope (unstable SAR_{local}); unscaled results are provided in Table S-5 and Fig. S-7.

References

1. ASTM International. *ASTM F2182-19e2: Standard Test Method for Measurement of Radio Frequency Induced Heating On or Near Passive Implants During Magnetic Resonance Imaging*, DOI: 10.1520/F2182-19E02 (2019).
2. Advanced Energy Industries, Inc. *LUXTRON M924 OEM MODULE* (2022).
